# Program evaluation of a pilot mobile developmental outreach clinic for autism spectrum disorder in Ontario

**DOI:** 10.1186/s12913-022-07789-7

**Published:** 2022-03-31

**Authors:** Mahdis Kamali, Shivajan Sivapalan, Anna Kata, Nicole Kim, Neshanth Shanmugalingam, Eric Duku, Lonnie Zwaigenbaum, Stelios Georgiades

**Affiliations:** 1 Department of Health Research Methods, Evidence and Impact, McMaster University, Hamilton, Canada; 2 Offord Centre for Child Studies, Hamilton, Canada; 3 SAAAC Autism Centre, Toronto, Canada; 4 Department of Psychiatry and Behavioural Neurosciences, McMaster University, Hamilton, Canada; 5 Autism Research Centre, Department of Pediatrics, University of Alberta, Edmonton, Canada; 6 McMaster Children’s Hospital, Hamilton, Canada

**Keywords:** Developmental disabilities, Autism spectrum disorder, Program evaluation, Cultural sensitivity, Early detection, Health services

## Abstract

**Background:**

Autism spectrum disorder (ASD) is a neurodevelopmental disorder with increasing prevalence worldwide. Early identification of ASD through developmental screening is critical for early intervention and improved behavioural outcomes in children. However due to long wait times, delays in diagnosis continue to occur, particularly among minority populations who are faced with existing barriers in access to care. A novel Mobile Developmental Outreach Clinic (M-DOC) was implemented to deliver culturally sensitive screening and assessment practices to increase access to developmental health services, reduce wait times in diagnoses, and aid in equitable access to intervention programs among vulnerable populations in Ontario.

**Methods:**

This study applied two evaluation frameworks (process and outcome evaluation) to determine whether the delivery model was implemented as intended, and if the program achieved its targeted goals. A mixed-methods design was undertaken to address the study objectives.

**Results:**

Between September 2018–February 2020, M-DOC reached 227 families with developmental health concerns for their child, while successfully targeting the intended population and achieving its goals. The mean age of the child-in-need at intake was 31.6 months (SD 9.9), and 70% of the sample were male. The program’s success was attributed to the use of cultural liaisons to break cultural and linguistic barriers, the creation of multiple points of access into the diagnosis pathway, and delivery of educational workshops in local communities to raise awareness and knowledge of autism spectrum disorder.

**Conclusions:**

The findings underscore the need for community-based intervention programs that focus on cultural barriers to accessing health services. The model of delivery of the M-DOC programs highlights the opportunity for other programs to adopt a similar mobile outreach clinic approach as a means to increase access to services, particularly in targeting hard-to-reach and vulnerable populations.

**Supplementary Information:**

The online version contains supplementary material available at 10.1186/s12913-022-07789-7.

## Introduction

Autism spectrum disorder (ASD) is a neurodevelopmental disorder defined by impairment in communication, social interaction, and behavioural development, combined with restrictive and repetitive behaviours, interests or activities [1]. Across the globe, prevalence of ASD is on the rise, impacting one in 160 children worldwide, notwithstanding underreporting in many low-and middle-income countries [51]. In Canada, one in 50 children aged 1–17 years have ASD and one in 44 children aged 8 years in the United States, with boys being 4 to 5 times more frequently diagnosed than girls [11, 35]. Observed increase in prevalence of ASD is linked to improved awareness and reporting, expansion of diagnostic criteria and more accurate diagnostic tools [51].

Developmental surveillance, screening and diagnosis are important for early identification of ASD, an integral element to early intervention and improved behavioral outcomes [3, 53, 54]. Diagnostic assessments typically involve both direct clinical observations and developmental interviews using standardized measures to inform clinical decision-making based on ASD criteria from the Diagnostic and Statistical Manual of Mental Disorders (DSM-5) [1]. In most jurisdictions, ASD diagnosis is required to access specialized interventions, shown to enhance cognitive and language abilities, and adaptive behaviours [17, 19, 37, 43]. However, delays in diagnosis continue to occur, especially amongst minority populations. In Ontario, families in need of ASD assessment and diagnosis are faced with long wait times, averaging 6–19 months from referral to receipt of diagnosis [33, 53, 54], causing additional barriers in access to care and early intervention among minority populations.

A study across six Canadian provinces found country of birth to be a significant factor in the age of diagnosis, noting children born outside of Canada were more likely to be diagnosed later than Canadian-born children [14]. A multitude of studies have also shown that children and families on the autism spectrum experience racial, socioeconomic and geographic inequities in both access to diagnostic services and care [15, 26, 29, 52]. Disparities in ASD assessment and access to care among these groups could be attributed to various factors, including cultural beliefs and acceptance of ASD, language barriers, negative experiences with the healthcare system, issues navigating multiple specialist services and restricted educational and/or financial resources [5, 28, 29, 45]. To address the identified barriers of access to care faced by minority groups, it is crucial to embed culturally-sensitive health care practices through the use of cultural liaisons to overcome linguistic barriers, provision of family-centered coordinated care, investment of time in developing trusting relationships and collection of information on patient satisfaction [16, 25, 31, 47]. By doing so, attitudes, behaviours and practices within healthcare can function appropriately, respectfully and responsive to culturally diverse patients, as well as in a way that is suitable to patient’s needs [27, 49].

In 2014, the largest survey of stakeholders of ASD in Canada, the 2014 *National Needs Assessment Survey* by Canadian Autism Spectrum Disorders Alliance (CASDA), helped develop a list of priorities aimed to improve surveillance and address service gaps in the country [46]. Timely access to ASD diagnosis and behavioural interventions was highlighted as a priority, as well as the need for targeted outreach to linguistically and culturally diverse communities in order to facilitate understanding of services and ultimately improve access to services. Subsequently, the provincial government created an Ontario Autism Program (OAP) Advisory Panel in 2019, appointing 20 distinct members, including parents of children on the autism spectrum, adults on the autism spectrum and experts from a range of various disciplines. The OAP made strong recommendations to the government to enhance cultural inclusivity and early intervention through an adoption of a ‘needs-based’ funding program [21]. Despite these efforts, many children and families in need remain on exceedingly long waitlists.

The South Asian Autism Awareness Centre (SAAAC), a non-governmental organization in Toronto, recognized the growing need for awareness, support and guidance for South Asian minority groups impacted by ASD. The organization’s mission is to make autism services equitable for all Canadians, by focusing on cultural elements South Asian communities face that may pose particular barriers, such as linguistic challenges, cultural stigma and misinformation regarding ASD [40]. This initiative is important as South Asian communities represent the largest visible minority group in Ontario, accounting for nearly one-third (30%) of the visible minority population and almost 9% of the province’s total population [41].

SAAAC responded to the needs of the community and call of action arising from the 2014 *National Needs Assessment Survey* by developing a mobile diagnostic assessment clinic for ASD. A novel Mobile Developmental Outreach Clinic (M-DOC) was implemented to deliver culturally sensitive screening and assessment practices in an effort to increase access to developmental health services, reduce wait times in ASD diagnoses, and ultimately aid in equitable access to intervention programs among vulnerable populations.

This study aims to apply a process and outcome evaluation framework to the M-DOC pilot program to determine whether the delivery model was implemented as intended, and if the program achieved its targeted goals. To our knowledge, this program evaluation is one of the first in Canada assessing the effectiveness of a mobile outreach clinic in the diagnosis and early intervention of ASD among ethnic minorities [23, 30, 34].

## Methods

SAAAC distributed a consent form to all patients and families included in the sample on the day of the initial screening assessment, and notified the families that information about their child will be used for quality improvement purposes by a McMaster evaluation team. Ethics approval for the evaluation of M-DOC pilot program was obtained through the Research Ethics Committee at McMaster University (Hamilton, ON).

### Study design

A formal summative (post-implementation) program evaluation was undertaken to evaluate the M-DOC pilot program. This is a systematic method of data collection and analysis of the activities, characteristics, and outcomes of programs in order to improve program effectiveness and inform future program development decisions [9]. Two evaluation frameworks were applied in this study: process evaluation and outcome evaluation. Process evaluation focuses on system-level and organizational characteristics of the implementation of a program and assesses whether the program was implemented as planned, with the best practice guidelines [42]. Outcome evaluation helped determine whether M-DOC achieved its goal and objectives by assessing the progress in the outcomes the program aimed to address [10]. A mixed-methods approach was undertaken inclusive of quantitative analysis and qualitative content analysis of survey data and participant feedback.

### Program activities

In September 2018, SAAAC partnered with the McMaster Autism Research Team (MacART) and local community organizations to develop and implement M-DOC. The pilot program was run between September 2018 and February 2020, delivering culturally sensitive screening and assessment practices in an effort to increase access to developmental health services, reduce wait times in ASD diagnoses, and ultimately aid in equitable access to intervention programs among vulnerable populations. The program was coined ‘mobile clinic’ since it overcame geographical barriers by selecting 10 accessible community locations (i.e. public schools, community centres, EarlyOn Centres) in five Neighbourhood Improvement Areas identified by the City of Toronto as high-need and underserved, to serve as clinic sites (Additional file 1 for a full list of site locations). This approach was undertaken as evidence suggests outreach targeted at community centres, advocacy organizations, schools and other community-based settings is an effective way to mobilize minority groups to access care who typically have troubles navigating through the complex healthcare system [5, 22].

In Ontario the pathway toward a diagnosis of ASD and post-diagnostic services typically begins with the parents of the child or their general practitioner flagging developmental concerns. The general practitioner will then refer to a pediatrician, who will either provide assessment or refer to a subspecialist. At this point, the pediatrician or subspecialist will diagnose the child on the autism spectrum, and the child can begin post-diagnostic services. Wait lists for these services are growing annually in Ontario, with wait times to receive an assessment from a specialist ranging from 1 year to 18 months [2, 6, 20]. The M-DOC pathway circumvents many of the roadblocks and wait times toward ASD diagnosis through a 3-step triage process (Additional file 2 for road map of typical Ontario pathway vs M-DOC).

First, a public education campaign was initiated at each of the M-DOC sites to inform parents and caregivers of atypical child development and warning signs of ASD, targeting families with children aged 18 to 72 months. Flyers regarding the information sessions were distributed by staff at the 10 centres, families were recruited via the flyers and word of mouth. These sessions promoted awareness of ASD, explained typical vs atypical development and addressed culturally associated misconceptions regarding developmental delays. After the session, parents and caregivers with concerns were encouraged to book an appointment at the clinic for screening of their child for ASD. Families that booked an assessment appointment returned to the clinic for the ‘Enhanced Developmental Screening’, which was done by a behavioural therapist and a general practitioner, who were both trained to administer the *Rapid Interactive (Screening) Test for Autism in Toddlers (RITA-T)* test [13], and *LookSee* checklists [7, 32]. The providers were fluent in both Tamil and English and able to provide direct translation for Tamil families, if needed. If a translator was needed for other languages, they were either a staff member at the centre, SAAAC staff or phone translation service. To ensure timeliness of the appointment, while the general practitioner collected a developmental history from parents and caregivers, the behavioural therapist observed the child and conducted screening activities from the RITA-T. The *LookSee* checklists were used as a Level 1 screening tool and only positive *LookSee* screens were then assessed using the RITA-T (details on the developmental screening tools can be found below). Following the appointment, the specialists consulted to review their findings prior to presenting the results of the screening to the families.

Children deemed low likelihood of ASD diagnosis were followed-up by the in-house case manager at 3 months, 6 months and if needed, 12 months to check whether the family had additional questions or concerns, or if the family had accessed other developmental services. If the child had a medium to high likelihood for ASD diagnosis, a referral was given for additional specialty services with a developmental pediatrician in the families’ area. The case manager followed-up with the family within 1–2 weeks to check-in on the family, provide additional resources and assist the family on booking an appointment with the referred developmental pediatrician. The case manager continued to follow-up with the family to check if they proceeded with the referral, whether the child received an ASD diagnosis or if the family accessed post-diagnostic services.

After the families’ initial visit, they were asked to complete a *Caregiver Satisfaction Survey *assessing their overall satisfaction with the quality of the care they received. A key feature of culturally sensitive health care is maintaining a patient centered relationship [31, 48, 49]. The implementation of the satisfaction survey in the program enabled an environment for the families to feel empowered to share their views on culturally sensitive health care. An additional advantage to this process is the notion that the health care providers are investing time in understanding the patient’s needs, while also displaying sensitivity and interest.

**Fig. 1:**
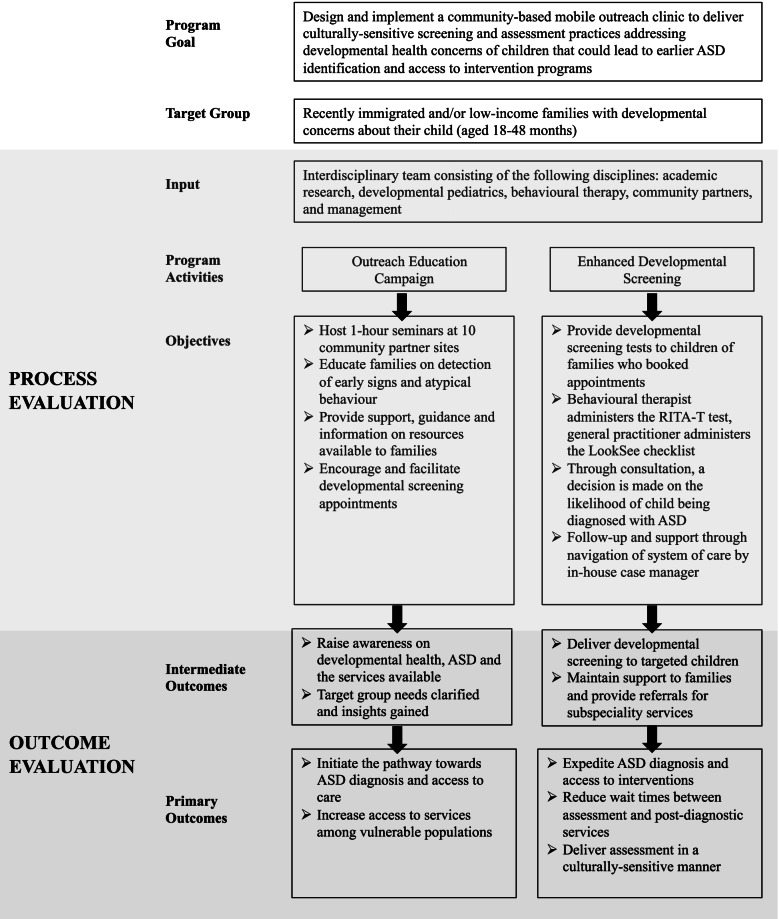
Logic Model of M-DOC Program Adapted from [23]

A logic model depicting the relationship between the M-DOC program components, processes and intended outcomes is displayed in Fig. 1 [50]**.** This road map of the program allows evaluation to highlight the link between program activities (cause) and outcomes (effect). The input refers to the resources, as in means necessary for the implementation of the program, and activities allude to the actions undertaken to bring about change for the individuals targeted [8]. Objectives are the direct result of the program activities, and outcomes are the anticipated changes that occur as a result of the inputs, activities, and outputs.

### Participants

A total of 153 families participated in the M-DOC program (Table 1). Male children comprised of 70% (*n* = 106) of the sample and the average age of the child-in-need at intake was 31.6 months (SD 9.9).

**Table 1:** Sociodemographic characteristics of program participants (*n* = 153)

Characteristic	N (%)
Sex	
Female	47 (31%)
Male	106 (69%)
Age (in months), mean (SD), min-max	31.6 (9.9),9–66
Number of siblings	
0	66 (43%)
1	68 (44%)
2	12 (8%)
3–5	7 (4.6%)
Parent relationship status	
Married/common-law	145 (95%)
Divorced/separated	7 (5%)
Single	1 (1%)
Household income, in CAD dollars	
0-35 K	67 (49%)
36-50 K	38 (28%)
51-75 K	19 (14%)
> 75 K	12 (9%)
Language spoken at home	
Tamil/English	30 (19.4%)
English	18 (11.7%)
Gujurati/English	10 (6.5%)
Tamil	9 (5.2%)
Bengali/English	5 (3.2%)
Other/Mixed	82 (52.3%)
Translator needed	
Yes	21 (14%)
No	132 (86%)

### Data sources and measures

The data sources that were used include meeting transcripts, dialogue with program staff, demographic and survey data, open-ended responses from surveys and phone interviews. These were chosen based on their suitability for measuring study outcomes and the quality of their psychometric properties.

#### Demographics

An *Intake Form Questionnaire* was administered to parents or caregivers at the first appointment. It included demographic information such as child’s age, parental occupation, number of siblings, family income, year of immigration to Canada and other demographic details.

#### Caregiver satisfaction and knowledge

The *Caregiver Satisfaction Survey,* developed for the purpose of the program evaluation, was administered to the child’s caregiver at the end of initial appointment (Additional file 3 for survey). It was designed to assess their perception of the quality of the service they received, overall satisfaction with the program, a self-evaluation of their knowledge of ASD and ability to navigate through health system, and an open-ended section to share additional feedback.

#### Screening tools

Two developmental assessment tools were administered to the children, the *Rapid Interactive (Screening) Test for Autism in Toddlers (RITA-T)* and *LookSee checklist.* The RITA-T is an interactive play-based Level 2 ASD screening tool that is validated and reliable for toddlers 18–36 months and designed to identify children who are likely to experience neurodevelopmental disorders in a low-risk population [12, 13]. It consists of 9 interactive activities that are known to indicate early signs of ASD. With a score for each of the 9 activities, the RITA-T total score can range from 9 to 30 with higher scores indicating greater symptom severity. Psychometric properties (at a cut-off score of > 14) indicate a sensitivity of 1.0, specificity of 0.84, and a positive predictive value (PPV) of 0.88 for identifying ASD likelihood in a high-risk group [12, 13, 18]. Categorization of likelihood of ASD in the M-DOC program included: low (RITA-T score less than 12), medium (RITA-T score between 13 and 15) and high (RITA-T score was above 16) [24]. Children deemed medium or high likelihood of ASD were referred to a developmental pediatrician for diagnosis. The *LookSee checklist,* formerly the Nipissing District Developmental Screening Tool, includes 13 checklists of a child’s developmental milestones in domains related to vision, hearing, speech, language, fine motor, gross motor, cognitive, social, emotional, and self-help [32]. Test-retest reliability is moderate with Spearman’s rank correlation at 0.61, *p* < 0.00 1[7].

#### Phone interviews

Follow-up calls were done by the in-house case manager after each appointment, and data was collected on the number of booked appointments, cancellations/no-shows, referrals, diagnosis information, and any additional feedback from the families.

### Evaluation framework

The process evaluation helped understand the outcome results by evaluating each process step in detail [39]. For the purpose of this study, process evaluation was structured into 3 main components: 1) implementation success; 2) context and characteristics; and 3) evaluation data collection process. Each process component was assessed by sub-questions and multiple indicators. The first component examined the delivery, fidelity, and acceptability of the M-DOC program. It aimed to determine the success rate of the selection process of the participants, and whether the program was delivered as intended. The second component addressed organizational factors of the program by assessing collaborative partnerships and community factors that could potentially impact program implementation. Finally, the last component allowed judgement of whether the appropriate outcome measures were selected to evaluate the program’s success and the completeness of the data collection. Additional file 4, Table 1 outlines the various components and elements of the process evaluation.

Outcome evaluation is considered an objectives-based approach as it assesses whether the goals of the program were met and the degree to which the program is having an effect on the target population [42]. To accurately appraise the impact of the M-DOC program, a baseline comparator is needed. Therefore, findings from SAAAC’s standard-of-care diagnostic screening services were used as a reference. This included 100 families surveyed at SAAAC who had undergone the ‘business-as-usual’ stream consisting of the typical Ontario diagnostic pathway described above. Socio-demographic sample characteristics are similar to the M-DOC program as they are all low-income, newcomer or immigrant families. Additional file 4 Table 2 outlines the evaluation goals, indicators and data sources needed to evaluate the effectiveness of the M-DOC program in meeting its objectives. The three main goals of the M-DOC program are: 1) increase access to developmental health services, 2) decrease wait times in receiving ASD diagnosis and early intervention, and 3) deliver culturally responsive practices to address developmental health concerns of families.

**Table 2:** Results of Process Evaluation

Process Measure	Evaluation Questions	Result
**1) Was the program implemented successfully?**		
RECRUITMENT & REACH	Was recruitment successful?	Of the 227 families that had booked appointments after the educational workshop, 169 (74%) attended screening appointment 16 families, or 9.5% (16/169) did not consent to the program, resulting in a final sample of 153 families
	Was the program offered to the intended target population?	107 children were born outside of Canada, 73% of children in the sample (107/146 with data) 77% of the families have an annual household income under $50,000 (105/136 with data) 14% of families needed a translator (21/153)
QUALITY	Are parents/caregivers satisfied with the program activities?	100% of families indicated ‘satisfied’ or ‘very satisfied’ on their overall experience of the M-DOC clinic on the Caregiver Satisfaction Survey
	Were services delivered in a high-quality manner?	3.2% (5/153) of families reported their appointment date was later than anticipated on the Caregiver Satisfaction Survey
**2) Which organizational factors impacted program implementation?**		
COLLABORATIVE PARTNERSHIPS	Was a collaborative advisory group created?	SAAAC Autism Centre collaborated with MacART, the Thorncliffe Collaborative for Muslim Families and Children organization, and community organizations (public schools, community centres, EarlyOn Centres)
CONTEXT	What were the contextual conditions of program implementation?	10 M-DOC clinic site locations selected on the basis of Neighbourhood Improvement Areas 42 educational workshop sessions held across site locations
**3) Was the acquisition of data successful?**		
COMPLETENESS	Is the evaluation data complete?	Mostly demographic indicators with missing data, 17% of annual household income and 28% of parental occupation missing
VALIDITY	Were the proper outcome measures used?	Post-screening, 92 referrals were made to a developmental pediatrician, which led to 84 children being diagnosed with ASD (91%)

## Results

Between September 2018 and February 2020, a total of 153 families participated in the M-DOC program (Table 1). Male children comprised of 70% (*n* = 106) of the sample and the average age of the child-in-need at intake was 31.6 months (SD 9.9). The majority of children had either no siblings or 1 sibling and came from married or common-law families (95%). Many families speak a mix of their cultural language and English at home, however Tamil/English was the most common languages spoken at home (19.4%).

### Process evaluation results

To assess whether the program was implemented successfully, the recruitment of families was evaluated, in addition to evaluating whether the program was offered to the intended target population (Table 2). Across the 10 community M-DOC sites from September 2018 to February 2020, 227 families booked screening appointments after attending the educational workshops. A total of 169 families (74%) attended the initial appointment, however 16 (9.5%) did not consent for subsequent screening, resulting in a final consented sample of 153 families.^1^

The program was targeted at newly immigrated or low-income families. Of the families with demographic data, almost three-fourths (73%, *n* = 107) were born outside of Canada and 77% (*n* = 105) had an annual household income of under $50,000 CAD. Almost all parents were married (94%) and a translator was needed for 21 families (14%).

In terms of quality of the program, whether the participants were satisfied with the program activities and that they were delivered in a high-quality manner was evaluated. A total of 100% of families reported being satisfied with their overall experience with the M-DOC clinic and only 5 families’ out of 153 said their appointment date was later than anticipated.

The implementation of the M-DOC program was made possible as a result of collaborative partnerships between SAAAC Autism Centre and distinct interdisciplinary teams. Collaborations with local community organizations were fostered to make cultural and linguistic liaisons between the families and SAAAC clinicians possible. SAAAC also partnered with an academic research group, the McMaster Autism Research Team (MacART), to develop and execute a research and evaluation program. Lastly, various community platforms were partnered with as site locations for diagnostic screening, including public schools, community centres, and EarlyOn Centres. The mobilization of screening and assessment capacity was made possible through a combination of utilizing in-house staff at SAAAC Autism Centre and external funding. SAAAC behavioural therapist and case manager provided in-kind contributions of their time for the program. External funding was used for outreach information sessions, research activities, incorporation of cultural liaisons and the implementation of level 2 screening.

In terms of the contextual factors that impacted program implementation, 10 site locations across the Greater Toronto Area in five Neighbourhood Improvement Areas were carefully selected as clinic sites in order to target ethnic and racially diverse communities. A total of 42 educational workshop sessions were held across the clinic sites, with the most (8 sessions) held at Morningside EarlyOn Child & Family Centre, Family Day Care Services at Scarborough EarlyOn Child & Family Centre, and Health Access Thorncliffe Park.

The completeness and validity of the data is the final portion of the process evaluation. Demographic variables were those with the highest percentage of missing data. Families were not comfortable disclosing their net annual household income and 17% of those data are missing. Similarly, information on parental occupation was sparse, with over one fourth of responses omitted (28%).

**Fig. 2:**
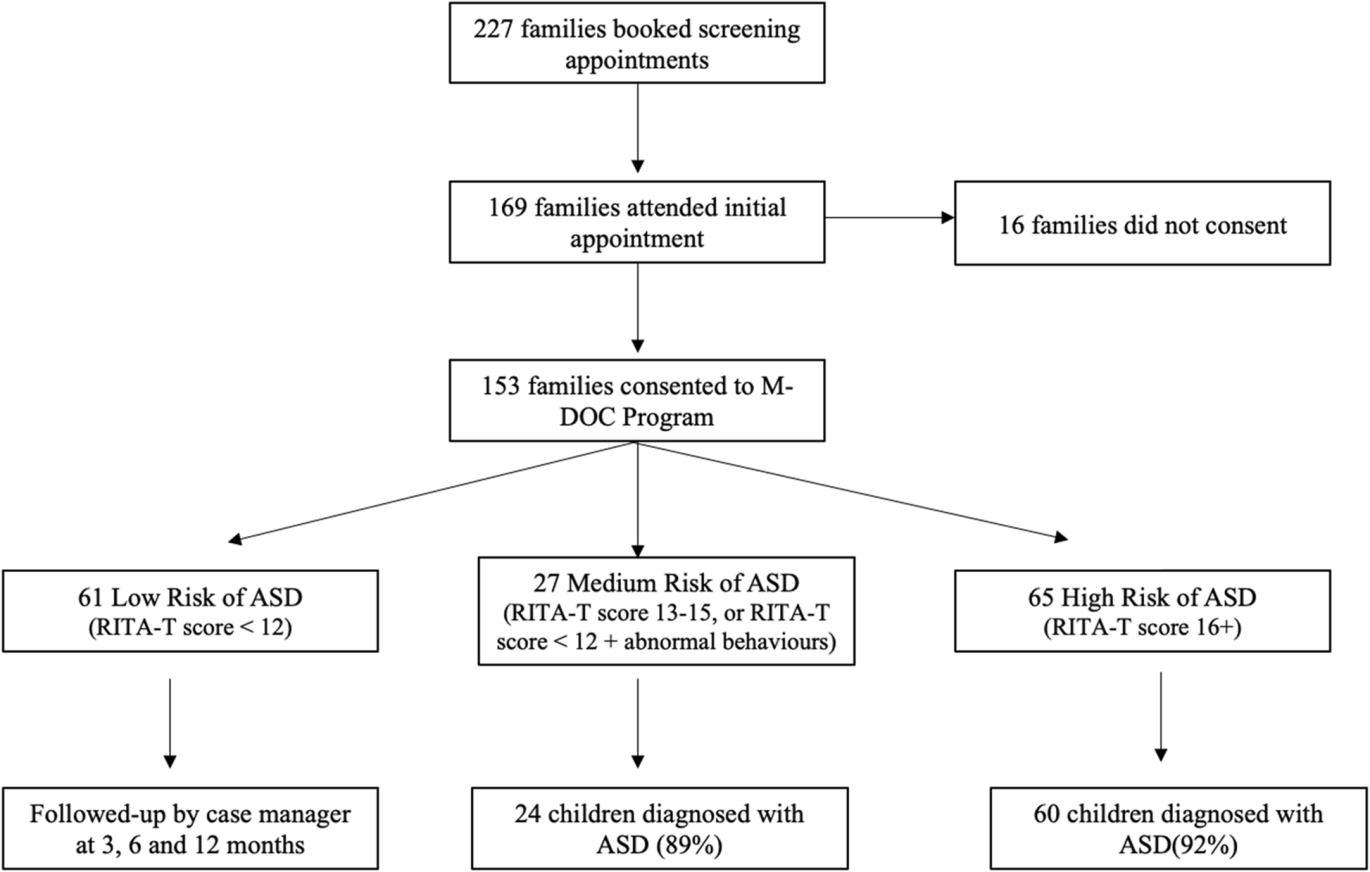
Flow chart

After the initial screening assessment using the RITA-T and *LookSee* checklists, a total of 92 referrals were made to developmental pediatricians in the families’ area of residence. Following the appointment from the specialist, 84 children were diagnosed as being on the ASD spectrum (Fig. 2). Likelihood of ASD was categorized as low for those children with a RITA-T score below 12, medium likelihood if their RITA-T score was between 13 and 15 or if they had a low RITA-T score but other atypical behaviour was observed, and high likelihood of ASD if their RITA-T score was above 16 [24]. The high proportion of diagnoses among children at medium (89%) and high likelihood (92%) of ASD points to the utility of RITA-T and *LookSee* in triaging families in need. Of 8 children that did not receive a diagnosis, 7 of them were lost to follow-up or did not attend final appointment to receive diagnosis.

### Outcome evaluation results

To assess whether access to developmental health services for newly immigrated and low-income families increased, the number of families that attended screening appointments was evaluated as well as the number and nature of the referral sources (Table 3). A total of 153 families attended developmental screening appointments and accessed services in the M-DOC pathway. Referrals to the program were made from a variety of sources, with the majority (89%, *n* = 136), from EarlyOn Centres developmental partners.

**Table 3:** Results of outcome evaluation

Evaluation questions	Results
***1) Was access to developmental health services increased?***	
Was access increased?	153 families accessed services in the M-DOC pathway
	136 of the referrals were made from developmental partners (EarlyOn Centres)
***2) Did wait times for ASD diagnosis and access to early interventions decrease?***	
Has the average age of diagnosis decreased?	**M-DOC program** (*n* = 58) average age of diagnosis = 35.8 months (20 months - 65 months), standard deviation=10.2
	**Standard-of-care pathway** (*n* = 56) average age of diagnosis = 66.7 months (24 months - 252 months), standard deviation =45.7
Has the average wait time to receive diagnosis decreased?	**M-DOC program** (*n* = 56) average wait time to diagnosis = 3.2 months (0.23 months - 8 months), standard deviation=1.74
	**Standard-of-care pathway** (*n* = 56) average wait time to diagnosis (n, %): 1-2 months (6, 10.7%) 3-6 months (19, 33.9%) 7 months – 1 year (10, 17.8%) 1-2 years (13, 23.2%) >2 years (8, 14.3%)
Has the average wait time to access post-diagnostic services decreased?	**M-DOC program** (*n* = 3) average wait time to services = 2.3 months (1 months - 4 months), standard deviation=1.53
	**Standard-of-care pathway** (*n* = 56) average wait time to services (n, %): 1-2 months (4, 7.1%) 3-6 months (10, 17.9%) 7 months – 1 year (14, 25.0%) 1-2 years (19, 33.9%) >2 years (9, 16.1%)
***3) Were services delivered in a culturally responsive manner?***	
Are parents/caregivers satisfied with the M-DOC program?	**M-DOC** (*n* = 153), ratings of overall satisfaction with M-DOC Program (n, %): Very dissatisfied (0, 0%) Dissatisfied (0, 0%) Neutral (0, 0%) Satisfied (15, 9.8%) Very Satisfied (138, 90.1%)
	**Standard-of-care pathway** (*n* = 56), ratings of satisfaction with diagnosis process (n, %): Very dissatisfied (10, 17.9%) Dissatisfied (11, 19.6%) Neutral (11, 19.6%) Satisfied (21, 37.5%) Very Satisfied (3, 5.4%)
	Ratings of satisfaction with receiving services process (n %): Very dissatisfied (14, 25.0%) Dissatisfied (10, 17.9%) Neutral (12, 21.4%) Satisfied (13, 23.2%) Very Satisfied (7, 12.5%)
	2 families dropped out of services
	58 families either cancelled their screening appointment or were no-shows
	Open-ended feedback from M-DOC program revealed families appreciated attending a local clinic in a familiar place, the follow-up calls, being offered services (workshops, support groups) during wait times
Do parents/caregivers perceive service providers are practicing cultural sensitivity?	Ratings of whether the assessment team carefully and respectfully explained the process and options in a way they understood (n, %): Yes (150, 98.0%) No (3, 2.0%)
	Ratings on being able to talk about everything they wanted to during the assessment (n, %): Very dissatisfied (0, 0%) Dissatisfied (0, 0%) Neutral (0, 0%) Satisfied (22, 14.4%) Very Satisfied (131, 85.6%)
	Ratings on feeling listened to by service provider (n, %): Very dissatisfied (0, 0%) Dissatisfied (0, 0%) Neutral (1, 0.6%) Satisfied (13, 8.5%) Very Satisfied (131, 90.1%)
	Ratings on feeling welcomed at the clinic Very dissatisfied (0, 0%) Dissatisfied (0, 0%) Neutral (0, 0%) Satisfied (12, 7.8%) Very Satisfied (141, 92.2%)
	Service providers often described as ‘helpful’, ‘informative’, ‘polite’, ‘patient’

Three distinct evaluation questions were posed comparing the M-DOC pathway to a standard-of-care as a counterfactual to assess whether the average age of diagnosis decreased in the M-DOC pathway, as well as the average wait time to receive diagnosis and access to services. The average age of diagnosis in the M-DOC pathway was 35.8 months (ranging from 20 months to 65 months; standard deviation (SD) = 10.2), compared to 66.7 months (ranging from 24 months to 252 months; SD = 45.7) in the standard-of-care pathway. On average, all families in the M-DOC program waited 3.2 months to receive a diagnosis, compared to only 10.7% of families in the standard-of-care pathway receiving a diagnosis in under 3 months. Once the families had received a diagnosis, those in the M-DOC program waited approximately 2 months to receive services, in contrast to those in the standard-of-care stream, where most families (75%) waited between 7 months to 2 years. These data and interpretations are made with caution due to small sample sizes.

In terms of satisfaction in accessing services, all 153 families indicated ‘satisfied’ or ‘very satisfied’ on their overall experience of the M-DOC clinic, compared to families in the standard-of-care pathway where 38% reported ‘very dissatisfied’ or ‘dissatisfied’ with diagnosis process with similar trends in satisfaction in receiving services post-diagnosis. A total of 153 families progressed within the M-DOC program, 2 families dropped out of services, and 58 cancelled their screening appointments or did not show up. A ‘drop out’ was considered when a family did not respond to 3 follow-up call attempts by the case manager. Information from open-ended feedback revealed that families in the M-DOC program appreciated being able to attend a local clinic in a familiar place. They also appreciated the follow-up calls and support from the case manager, and being offered services in the form of educational workshops and support groups during wait times for diagnosis and assessment. All indicators assessing whether families perceived service providers as practicing with a culturally sensitive approach were rated favourably. Parents and caregivers believed the assessment team carefully and respectfully explained the process and options in a way they understood, they were able to talk about everything they wanted to during the appointments, they felt listened to by service providers and welcomed at the clinic. They described the service providers as ‘helpful’, ‘informative’, ‘polite’ and ‘patient’; full excerpts can be found in Additional file 5.

## Discussion

This is the first known study to evaluate the process and outcomes of a mobile developmental outreach clinic for ASD in Canada. It assessed the implementation and coverage of a novel model of delivery for immigrant and low-income families with developmental health concerns for their child. Unique features of the program include its targeted approach to serve families locally in the community, the use of cultural liaisons to break cultural and linguistic barriers, multiple points of access into the pathway, and delivery of educational workshops to raise awareness and knowledge.

The evaluation revealed that, between September 2018 and February 2020, the program was able to reach 227 families with developmental health concerns for their child while successfully targeting the intended population, as the majority of families were immigrants and low-income. The collaborations and partnerships among community centres, public schools, EarlyOn centres and linguistic liaisons not only allowed for the physical implementation of 10 outreach clinics and 42 educational workshops, but also enriched the program’s reach through referrals and accessibility, as the M-DOC clinic sites were located in areas that facilitated access for targeted families. The RITA-T and *LookSee* checklists proved to be highly accurate in detecting developmental delays and ASD, with 91% of children who were referred to a specialist received an eventual diagnosis of ASD.

Results from the outcome evaluation showed access to developmental health services were made possible for 153 families, of which 136 were referred from a developmental partner organization, namely the EarlyOn centres. An important achievement of the program includes the striking decrease in age of diagnosis, wait time to receive diagnosis and access to services compared to the standard-of-care pathway. Additionally, parents and caregivers were deeply satisfied with the M-DOC program and believed service providers exhibited culturally sensitive practices to address their developmental health concerns.

Inferences from the findings of this work can be used to benefit various populations across Canada. The success of the M-DOC programs highlights the opportunity for other programs to adopt a similar mobile outreach clinic approach as a means to increase access to services. This model of delivery is particularly advantageous in targeting hard-to-reach and vulnerable populations, who face barriers in accessing care in terms of cultural stigma, discrimination, language barriers, and health care system illiteracy [4, 25, 36, 38]. This study also underscores the importance of collaborations and partnerships. Without buy-in from the local community and partners, the implementation and execution of the program wouldn’t be possible. This collaboration is necessary to promote the capacity building of an integrated program that is targeted, inclusive and where resources can be shared [22].

The success of the M-DOC program demonstrates the possibility of a substantial reduction in wait times to receive an ASD diagnosis and subsequent access to early intervention. Such improvement is crucial as it is known that early intervention leads to improvements in cognitive and language abilities, and adaptive behaviours in children on the autism spectrum [17, 19, 37, 43]. However, sustainability is flagged as a concern since the program would not be able to operate without the financial help of non-governmental organizations, government support and funders. This notion, coupled with the mounting evidence of racial, socioeconomic, and geographic inequities in ASD assessment and access to care substantiate the need for the Canadian provincial and federal governments develop distinct practice guidelines along with funding for minority groups [15, 26, 29, 52]. Doing so will ensure that access to care is truly universal for populations all across the country.

It has been shown that the M-DOC program was successful in implementing program activities as planned and achieving its goals. In order to further evaluate the characteristics and outcomes of the program, improve program effectiveness and to inform decisions about future programming, the following recommendations are made. In terms of data acquisition, it would be useful to collect information on the costs of the program in order to conduct an economic evaluation to analyze whether the benefits of the program outweighed the costs compared to the standard-of-care pathway [44]. While the data in this study presented compelling evidence for the program’s success in terms of reduction in wait times, improvements in access and family satisfaction of cultural responsiveness, it is important to measure whether these changes are sustained over time. Future work could collect data longitudinally from families along the pathway to evaluate their satisfaction with services at multiple time points. Additionally, it was observed that few families dropped out of services, cancelled appointments or were no shows; however, information surrounding their reasoning was not collected. It would be useful to probe these parents/caregivers to further identify barriers that they may have faced. By understanding the difficulties that families bear, program developers could tailor activities to be more conducive to accessibility. Given that this pilot program did not undertake an experimental design, future research could use such approach in order to test effectiveness of assessment tools and program activities. Lastly, research on the implementation and outcomes of a diagnostic screening program for ASD is complex and multi-dimensional. In the future, perceptions of stakeholders at different levels could be collected. Surveys could be administered to staff and project partners to capture their view on program activities, barriers and facilitators and opportunities for improvement.

SAAAC responded to the needs of the Ontario Autism Program, CASDA’s 2014 *National Needs Assessment Survey*, and vulnerable populations in need by targeting outreach to linguistically and culturally diverse communities as a means to facilitate understanding and access to ASD services. The findings from this study confirm the M-DOC pilot program’s success in terms of implementation and goals achieved, and learnings can be applied unilaterally to inform other programs, policy and practice guidelines.

## Supplementary Information

**Additional file 1.**

**Additional file 2.**

**Additional file 3.**

**Additional file 4.**

**Additional file 5.**

## Data Availability

The datasets generated and analysed during the current study are not publicly available due confidentially and ethical guidelines but are available from the corresponding author upon reasonable request.

## Abbreviations

ASD: Autism Spectrum Disorder
CASDA: Canadian Autism Spectrum Disorders Alliance
DSM: Diagnostic and Statistical Manual of Mental Disorders
M-DOC: Mobile Developmental Outreach Clinic
MacART: McMaster Autism Research Team
OAP: Ontario Autism Program
PPV: Positive Predictive Value
RITA-T: Rapid Interactive Test for Autism in Toddlers
SAAAC: South Asian Autism Awareness Centre
SD: Standard Deviation

## Footnotes

1 It is important to note that 11 of the 16 families who did not consent were from the Health Access Thorncliffe Park (HATP) location where research was already underway, and families had consented to HATP’s intake process and thus likely hesitant to consent again; therefore they were not included in this project.
