# Supplementary material for: Program evaluation of a pilot mobile developmental outreach clinic for autism spectrum disorder in Ontario
Source: BMC Health Serv Res. 2022 Mar 31;22:426. doi: 10.1186/s12913-022-07789-7 (PMC8973535; doi:10.1186/s12913-022-07789-7)
Supplement: Supplementary file 1 — Additional file 1. [file 12913_2022_7789_MOESM1_ESM.docx]

Additional file 1: Project Locations

The 10 M-DOC site locations included:

1. SAAAC Autism Centre [9127-Woburn]
2. West Scarborough Neighbourhood Community Centre [117-L’Amoreux]
3. Morningside Early ON Child & Family Centre [136-West Hill]
4. Family Day Care Services at Scarborough Early ON Child & Family Centre [137-Woburn]
5. The West Scarborough Neighbourhood Community Centre [124- Kennedy Park]
6. H.A Halbert Junior Public School [121- Oakridge]
7. West Scarborough Neighbourhood Community Centre [121- Oakridge]
8. Thorncliffe Public School [55- Thorncliffe Park]
9. Jenner Jean-Marie Community Centre [55- Thorncliffe Park]
10. Health Access Thorncliffe Park [55- Thorncliffe Park]
