# Supplementary figures and images for: Program evaluation of a pilot mobile developmental outreach clinic for autism spectrum disorder in Ontario
Source: BMC Health Serv Res. 2022 Mar 31;22:426. doi: 10.1186/s12913-022-07789-7 (PMC8973535; doi:10.1186/s12913-022-07789-7)

Additional file 2: Comparison of Typical Ontario Pathway and M-DOC Pathway

**
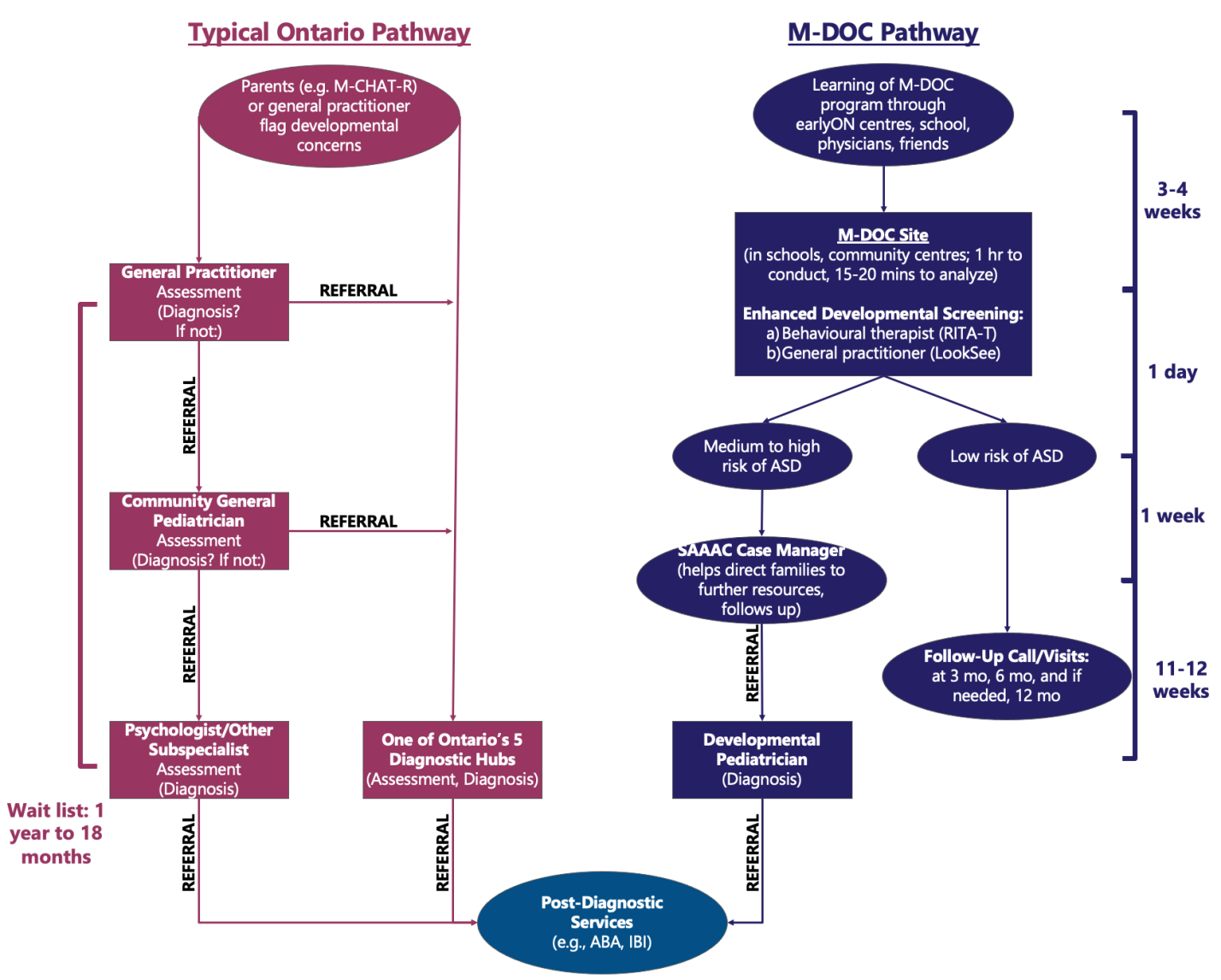
**

Supplement: Supplementary file 2 — Additional file 2. [file 12913_2022_7789_MOESM2_ESM.docx]
