# Supplementary material for: Program evaluation of a pilot mobile developmental outreach clinic for autism spectrum disorder in Ontario
Source: BMC Health Serv Res. 2022 Mar 31;22:426. doi: 10.1186/s12913-022-07789-7 (PMC8973535; doi:10.1186/s12913-022-07789-7)

**SAAAC Autism Centre - MDOC**

# Caregiver Satisfaction Survey

*Please take a few minutes to fill out this survey on the timeliness and quality of the service you received today. We want to assess your current experiences and knowledge coming into this process so that we can see the difference this process has made in impacting the development of your child and yourselves.*

## BACKGROUND

### **Has your child been assessed/screened for any special needs or related disorders?**

◻ YES **IF YES, PLEASE SPECIFY:**

◻ NO

### **How many times have you visited** [Healthcare facility name] **within the past year?**

◻ 1 TIME ◻ 2-4 TIMES ◻ MORE THAN 5 TIMES

## QUALITY OF SRVICE

### **Was your appointment date later than you expected?**

◻ YES

◻ NO

### **Did the assessment team carefully and respectfully explain the process and your options in an** in a way that you understood**?**

◻ YES

◻ NO

## SELF- EVALUATION

### **How would you rate your knowledge about Typical/Atypical Development?**

|  |  | ◻ VERY POOR | ◻POOR | ◻ FAIR | ◻GOOD | ◻ VERY GOOD |  |  |  |
| --- | --- | --- | --- | --- | --- | --- | --- | --- | --- |

### **How would you rate your current level of stress/anxiety when thinking about your child’s development?**

| ◻ VERY STRESSED | ◻ STRESSED ◻ NEUTRAL | ◻ A LITTLE BIT STRESSED ◻ NOT STRESSED |
| --- | --- | --- |

### **How would you rate your ability to navigate in the community for resources for your child?**
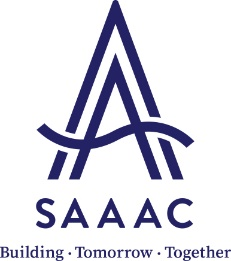


| ◻ VERY POOR | ◻ POOR | ◻ FAIR ◻ GOOD ◻ VERY GOOD |
| --- | --- | --- |

## ADDITIONAL FEEDBACK

| **PLEASE SHARE ANY ADDITIONAL COMMENTS:** |
| --- |
|  |
|  |
|  |
|  |
|  |
|  |

## OVERALL RATING

### How would you rate your overall satisfaction with…

|  | Very Dissatisfied | Dissatisfied | Neutral | Satisfied | Very Satisfied |
| --- | --- | --- | --- | --- | --- |
| Your experience with the MDOC |  |  |  |  |  |
| Getting to talk about everything you wanted to today |  |  |  |  |  |
| Feeling listened to by the doctor/behaviour therapist |  |  |  |  |  |
| Feeling welcomed at the clinic |  |  |  |  |  |

***Thank you for taking the time to fill out our survey. We rely on your feedback to help us improve our services. Your input is greatly appreciated.***
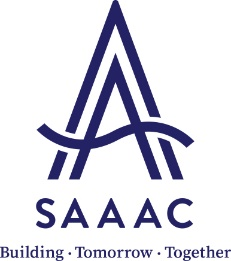

Supplement: Supplementary file 3 — Additional file 3. [file 12913_2022_7789_MOESM3_ESM.docx]
