# Supplementary material for: Program evaluation of a pilot mobile developmental outreach clinic for autism spectrum disorder in Ontario
Source: BMC Health Serv Res. 2022 Mar 31;22:426. doi: 10.1186/s12913-022-07789-7 (PMC8973535; doi:10.1186/s12913-022-07789-7)
Supplement: Supplementary file 4 — Additional file 4. [file 12913_2022_7789_MOESM4_ESM.docx]

Additional file 4: Process and Outcome Evaluation Matrix

Table 1: Process Evaluation Matrix

| **Process Measure** | **Evaluation Questions** | **Process Indicators** | **Data Source** | **Timing of Data Collection** | **Analysis Plan** |
| --- | --- | --- | --- | --- | --- |
| **1) Was the program implemented successfully?** | | | | | |
| RECRUITMENT & REACH | Was recruitment successful? | Proportion of families who attended screening appointment/families that booked appointment after education workshop  Numbers and percentage of families who did not sign consent | Document review | Ongoing | Proportions |
|  | Was the program offered to the intended target population? | Demographic characteristics of the study population | Intake Form Questionnaire | Before initial appointment | Proportions, frequencies |
| QUALITY | Are parents/caregivers satisfied with the program activities? | Numbers and percentage of caregivers that indicated ‘satisfied’ or ‘very satisfied’ on their overall experience of the M-DOC clinic  Open-ended feedback | Caregiver Satisfaction Survey | After screening appointment | Proportions, frequencies, content |
|  | Were services delivered in a high-quality manner? | Numbers and percentage of caregivers that indicated ‘yes’ to their appointment date being later than expected | Caregiver Satisfaction Survey | After screening appointment | Proportions, frequencies |
| **2) Which organizational factors impacted program implementation?** | | | | | |
| COLLABORATIVE PARTNERSHIPS | Was a collaborative advisory group created? | Number of members  Proportion of partners engaged in activities | Document review | Ongoing | Content |
| CONTEXT | What were the contextual conditions of program implementation? | Information on the M-DOC clinic site locations  Number of educational workshop sessions | Document review | Ongoing | Content |
| **3) Was the acquisition of data successful?** | | | | | |
| COMPLETENESS | Is the evaluation data complete? | Percentage of missing data  Characteristics of missing data | Intake Form Questionnaire, Caregiver Satisfaction Survey, follow-up calls | After screening appointment | Proportions, content |
| VALIDITY | Were the proper outcome measures used? | Number of referrals to specialty services that led to a diagnosis  Feedback from diagnostic test assessors | Document review, follow-up calls, dialogue | 1, 3, 6, 12 months after developmental screening | Frequencies |

Table 2: Outcome Evaluation Matrix

| **Evaluation Questions** | **Outcome Indicators** | **Data Source** | **Timing of Data Collection** | **Analysis Plan** |
| --- | --- | --- | --- | --- |
| **GOAL 1: Increase access to developmental health services for newly immigrated and/or low-income families** | | | | |
| Was access increased? | Numbers of families that attended screening appointment    Number and nature of referral sources | Intake Form Questionnaire | Before initial appointment | Count |
| **GOAL 2: Decrease in wait times in receiving ASD diagnosis and early intervention** | | | | |
| Has the average age of diagnosis decreased? | Age of diagnosis in M-DOC program  Age of diagnosis in standard-of-care pathway | Follow-up calls, standard-of-care survey | 1, 3, 6, 12 months after developmental screening | Tabulate |
| Has the average wait time to receive diagnosis decreased? | Time waiting for diagnosis in M-DOC program  Time waiting for diagnosis standard-of-care pathway | Follow-up calls, standard-of-care survey | 1, 3, 6, 12 months after developmental screening | Tabulate |
| Has the average wait time to access post-diagnostic services decreased? | Time waiting for services in M-DOC program  Time waiting for services in standard-of-care pathway | Follow-up calls, standard-of-care survey | 1, 3, 6, 12 months after developmental screening | Tabulate |
| **GOAL 3: Deliver culturally sensitive practices to address developmental health concerns of families** | | | | |
| Are parents/caregivers satisfied with the M-DOC program? | Numbers and percentage of caregivers that indicated ‘satisfied’ or ‘very satisfied’ on their overall experience of the M-DOC clinic  Numbers and percentage of caregivers that indicated ‘satisfied’ or ‘very satisfied’ on their overall experience of the standard-of-care pathway  Number of families who have dropped out of services  Number of no-shows/cancellations for M-DOC appointment  Open-ended feedback | Caregiver Satisfaction Survey, standard-of-care survey, document review, follow-up calls | After screening appointment | Proportions, frequencies, content |
| Do parents/caregivers perceive service providers are practicing cultural sensitivity? | Numbers and percentage of caregivers that indicated ‘yes’ to the assessment team carefully and respectfully explaining the process and options in a way they understood  Numbers and percentage of caregivers that indicated ‘satisfied’ or ‘very satisfied’ on being able to talk about everything they wanted  Numbers and percentage of caregivers that indicated ‘satisfied’ or ‘very satisfied’ on being listened to by service provider  Numbers and percentage of caregivers that indicated ‘satisfied’ or ‘very satisfied’ on feeling welcomed at the clinic  Open-ended feedback | Caregiver Satisfaction Survey | Proportions, frequencies, content | Proportions, frequencies, content |
