# Supplementary material for: Program evaluation of a pilot mobile developmental outreach clinic for autism spectrum disorder in Ontario
Source: BMC Health Serv Res. 2022 Mar 31;22:426. doi: 10.1186/s12913-022-07789-7 (PMC8973535; doi:10.1186/s12913-022-07789-7)
Supplement: Supplementary file 5 — Additional file 5. [file 12913_2022_7789_MOESM5_ESM.docx]

Additional file 5: Open-ended Feedback from Parents/Caregivers

1. A great opportunity to know the development and needs of my child.
2. Both of the service provider were very welcomed and they explained everything with good details. They were very nice and open about the signs at the same time they were with very positive attitude.
3. Clear, precise and informative.
4. Doctors were excellent, helpful, informative. Very polite & explained in a very simple manner.
5. Dr. XX and XX were very helpful, glad I saw them today. They help me with my concern answered in very polite and softly especially in this stressful situation. Thank you so much for helping me.
6. Everything was good in the appointment. Happy with the appointment.
7. Excellent assessment and feedback
8. Excellent service, listened to my concern with interest. Answered all of my repeated questions and concerns. Received very positive feedback and solutions. Thank you.
9. Helpful and great experience. Keep up the good work. It helps a lot of families.
10. I just came today & have the opportunity to meet the MDOC. I am very satisfied to get their appointment so early.
11. I just can't express how happy and relieved I am after my visit. It was very informative and helpful and took my stress away.
12. It was a good assessment and info.
13. It was very helpful and good to understand the child's behaviour. The doctors were very patient and explained everything clearly.
14. Thank you for this mobile service. Wonderful doctor and therapist. Excellent.
15. The information we received was helpful. We appreciate the feedback.
16. The people who assessed my son was very nice.
17. The team assessed for my child in a way that I understood. They explained very clearly about her problems. They tested her through variety of ways, and talked me to do about next steps. I had a very good information about my child for future steps. Thank you very much for them.
18. The therapists were very professional and made us feel welcomed. They carried out the assessment perfectly and made sure we were involved throughout the whole process.
19. Very experienced and knowledgeable.
20. Very friendly and put my mind at ease.
21. Very informative.
22. Very patient and understanding.
23. Very smart doctor and therapist.
24. Very useful information.
25. We are very glad to meet them and thank you so much for their concern about my child. They both really doing great service and hope to get their assistance furthermore.
26. We got to know about our son's progress. He didn’t have any test before. We would love to have more screening in the future.
